# *Leea macrophylla* Roxb. leaf extract potentially helps normalize islet of β‐cells damaged in STZ‐induced albino rats

**DOI:** 10.1002/fsn3.625

**Published:** 2018-03-30

**Authors:** Md. Atiar Rahman, J. M. Kamirul Hasan Chowdhury, Jannatul Aklima, Mohammad Ali Azadi

**Affiliations:** ^1^ Department of Biochemistry & Molecular Biology University of Chittagong Chittagong Bangladesh; ^2^ Department of Zoology University of Chittagong Chittagong Bangladesh

**Keywords:** albino rats, *Leea macrophylla*, polyphenols, streptozotocin, type2 diabetes mellitus

## Abstract

This research aims to investigate the protective effects *Leea macrophylla* Roxb polyphenols on streptozotocin‐induced diabetic rats. Polyphenolic assays were undertaken through established methods. To conduct animal intervention study, forty Wistar albino male rats (average body weight 188.42 ± 7.13 g) of different groups were diabetized by streptozotocin (60 mg/kg) only in the animals of diabetic control (DC) and *L. macrophylla* extract (LM) groups. At the end of 4 weeks of intervention, serum was analyzed for insulin, liver and cardiac enzymes, lipid profiles, uric acid, and creatinine using ELISA method. In vitro α‐amylase inhibition of LM was evaluated and compared with reference drug acarbose. Pancreatic tissues were undertaken for histopathological screening. Food and fluid intake, weekly blood glucose level, liver glycogen, aspartate transaminase (AST), creatinine kinase (CK‐MB), cholesterol, and lactate dehydrogenase (LDH) were significantly decreased, whereas oral glucose tolerance (OGTT) ability, serum insulin concentration, and pancreatic islets morphology were significantly improved in the LM300 treatment group compared to the DC group. Alpha‐amylase inhibition was not found to be very promising for guiding the α‐amylase inhibition pathway. Results suggest that *L. macrophylla* can exert a potential effort to restore pancreatic β‐cell damaged by streptozotocin induction.

## 1 INTRODUCTION

Diabetes mellitus (DM) is a major health problem causing morbidity and mortality worldwide, and it is the most chronic disease characterized by high blood glucose level. It is one of the complex metabolic disorders, and it is considered as one of the major risk factors for cardiac diseases, liver dysfunction, and dystrophy in skeletal muscle and adipose tissue (Eckel, Grundy, & Zimmet, 2005; Grundy, 2006; Pooya et al., 2010). The International Diabetes Federation (IDF) reported that the worldwide prevalence of diabetes afflicted 366 million people in 2011, and the number is expected to rise to 552 million by 2030. Therapeutic options of DM especially focus on glycemic control involving regular exercise, controlled diet, oral antihyperglycemic drugs, and exogenous insulin administration (Fitzer & de la Torre, 2012; Moser, Morris, & Garg, 2012). These monotherapeutics or combinations of antidiabetic agents sometimes become ineffective to control diabetic complications and show major side effects. Sulfonylurea and biguanides, for example, are very expensive, and they have undesirable side effects or contraindication (Chen, Li, Sun, & Ma, 2013; Halim Eshrat, Hussain, Jam, & Rao, 2001). Sulfonylurea shows some common side effects such as heartburn, vomiting, and skin rashes (Panneerselvam, 2004), while the Biguanides (metformin) can cause gastrointestinal discomforts, anorexia, vomiting, and B_12_ malabsorption on long‐term consumption. Therefore, it is important to search for more effective and safer antidiabetic agents. In this study, we reported the prospective uses of *L. macrophylla* in normalizing the streptozotocin‐induced β‐cell damage in an animal intervention therapy.

*Leea macrophylla*, belonging to Leeaceae family, was identified as an Indian habitat, and it is known as Hastikarnapalasa (Kangale, 2006; Singh & Singh, 1981). It is also known as Dholsamudra, Hathikana, or more commonly Hatikana (Elephant's ear), which might be named from the size and shape of its leaf looking like an Elephant's ear. Apart from its availability in North Indian area, it is distributed to Nepal, Cambodia, Laos, Myanmar, Thailand, Eastwards of Ganges, Bihar, Bengal, Assam, the Terai, and western India as a herb or herbaceous shrub (Al Faruq et al., 2014). In Bangladesh, it is distributed in Rajshahi, Savar, Jessore, some parts of Chittagong Hill tracts, and rarely in Dinajpur. An Ethnopharmacological use of this plant is documented for urinary problem by local tribes of Bihar (Hains, 1961). Ethnobotanically, the leaves have been used in goiter, gastric tumor, lipoma, and tetanus (Uddin, 2006). Some other tribes use the leaf as vegetables (Jadhao, Wadekar, & Mahalkar, 2009). Crude and powder of leaves are traditionally used in cancer, urolithiasis, wounds and sores, goiter, gastric, tumor, tetanus, and urinary disturbances (Garodia, Ichikawa, Malani, Sethi, & Aggarwal, 2007; Nizami, Rahman, Ahmed, & Islam, 2012; Swarnkar & Katewa, 2008). Leaf juice is also used as an anti‐inflammatory agent in boils, arthritis, gout, and rheumatism (Dewanjee, Dua, & Sahu, 2013; Uddin, Hassan, & Sultana, 2006). It is also applied externally to allay pain and to stop the effusion of blood (Zaoui et al., 2002). Leaf extracts are reported as hepatoprotective, antiamnesic, and neuroprotective (Ferdousy et al. 2016). These are extensively used by the ayurvedic physicians in the preparation of seasonal tonic modaka (Singh & Singh, 1981). The dried root powder mixed with clarified butter is prescribed in the morning as age sustainer (Jadhao et al., 2009). Very recently, its antioxidative effect, which is considered as the pivotal functions for pathophysiological repairing, has been reported and plant phenolics, saponin, glycoside, carbohydrate, and protein types of compounds were revealed in the phytochemical studies with the seed extracts of *L. macrophylla* (Akhter, Rahman, Aklima, Hasan, & Hasan Chowdhury, 2015; Islam et al., 2009). Hypothetically, the antioxidative potential afflicts to have a higher involvement in reducing diabetic complications. However, no study has been conducted to scientifically report whether *L. macrophylla* has antidiabetic effects. Thus, this research aims to evaluate the antidiabetic potentiates of *L. macrophylla* streptozotocin‐induced animal model.

## 2 MATERIALS AND METHODS

### 2.1 Collection and identification of plant

*Leea macrophylla* (Hatikana) leaves were collected from Bangladesh Council of Scientific and Industrial Research, Rajshahi Centre, Bangladesh. The plant was taxonomically confirmed by Dr. Sheikh Bokhtear Uddin, Taxonomist and Professor, Department of Botany, University of Chittagong. A sample specimen of the plant has been preserved both in the Departmental Herbarium and online database with the accession number ACCU‐2011/07.

### 2.2 Chemicals and reagents

Streptozotocin was used as a diabetogenic agent in this experiment. It was procured from Sigma‐Aldrich Chemical company (Lot # SLBH0076V). Folin–Ciocalteu reagent and quercetin were procured from Sigma‐Aldrich chemicals (St. Louis, MO, USA). All other chemicals and reagents used in this study were of analytical grade until unless specified individually.

### 2.3 Preparation of plant extract

Shade‐dried fresh leaves of *L. macrophylla* were grounded into a fine powder (800 g) and extracted with 98.5% ethanol for 10 days at room temperature with occasional stirring. The extract was then filtered off through a filter paper and evaporated at 43–45°C under reduced pressure through a rotary evaporator (RE200; Biby Sterilin, UK) to have a dry residue (44.8 g, yield 5.6%).The whole extraction process was repeated three times, and finally, concentrated extract was collected to preserve in the refrigerator at 4°C.

### 2.4 Determination of total phenolic content (TPC)

Total phenolic content of *L. macrophylla* extract was spectrophotometrically determined at 765 nm using Folin–Ciocalteu method described by Chang et al. (2002) with minor modification. A standard calibration curve was prepared using different concentrations of gallic acid (1–16 μg/ml). Leaf extract was prepared in ethanol at a concentration of 20 mg/ml. A 20 μl sample or 20 μl standard solution was taken in screw cap tube and added 1.58 ml distilled water to the tube. After that, 100 μl FC reagent was added and incubated at room temperature for 1–8 min. Then, 300 μl Na_2_CO_3_ (20%) solution was added into the tube and incubated at room temperature for 2 hr. The total phenolic content was calculated as gallic acid equivalent (GAE) by the following equation:

$$C=\frac{\left(c\times V\right)}{m};$$

where C = TPC (mg/g plant extract in GAE), c = concentration of sample obtained from calibration curve (mg/ml), V = volume of the sample, and m = sample weight (g).

### 2.5 Determination of total flavonoid content (TFC)

Total flavonoid content (TFC) of *L. macrophylla* (LM) was spectrophotometrically determined at 415 nm according to the method established by Kumaran and Karunakaran (2007). Quercetin from Sigma‐Aldrich Chemicals Ltd. (USA) was used, and a standard calibration curve was prepared using different concentrations (12.5–200 μg/ml). Leaf extract was prepared in ethanol at a concentration of 2 mg/10 ml. At first, 1 ml extract or 1 ml standard solution was taken in a screw cap tube and then added 3 ml methanol into the tube. After that, 200 μl 10% AlCl_3_ and 200 μl 1 M CH_3_COOK were added to the tube. Finally, 5.6 ml distilled H_2_O was added and incubation at room temperature for 30 min. The total flavonoid content was calculated through the following equation:

$$C=\frac{\left(c\times V\right)}{m};$$

where C = TFC (mg/g plant extract in quercetin), c = concentration of sample obtained from calibration curve (mg/ml), V = volume of the sample, and m = sample weight (g).

### 2.6 Animals

Six‐ to 7‐week‐old male Wistar albino rats with mean BW 188.42 ± 7.13 g were procured from the Bangladesh Council of Scientific and Industrial Research (BCSIR), Chittagong‐4220, Bangladesh. The animals were housed as two in one medium‐sized polycarbonated cage in a temperature and humidity‐controlled room (temperature 22 ± 1°C and humidity 55%–60%) with a 12‐hr light–dark cycle. All animals were fed with a commercial rat pellet diet during the entire experimental period. Animals were handled and maintained according to the local animal ethical guidelines approved by the institutional Animal Ethics Review Board (Ethical approval AERB/FBS/UC/01, 2015) of the Faculty of Biological Sciences, University of Chittagong.

### 2.7 Toxicity test and dose fixation

Five animals maintained in standard laboratory condition were used for toxicity study to fix the dose for intervention. The animals received a single dose of 0.5, 1.0, 1.5, and 2.0 g/kg BW of LM orally. Animals were kept overnight fasting prior to administration the LM. Once the dose was administered, food was withheld for next 3–4 hr. Animals were individually kept in close observation during the first 30 min after dosing, periodically first 24 hr (special attention for the first 4 hr), thereafter for a period of 3 days to record the delayed toxicity. Cage side was observed once daily for recording the changes in eyes and mucous membrane, skin and fur, respiratory and circulatory rate, and autonomic and CNS system. Effective therapeutic dose fixed as one‐tenth of the median lethal dose (LD_50_ > 2.0 g/kg) (Zaoui et al., 2002).

### 2.8 Plant sample preparation for intervention

*Leea macrophylla* samples were prepared from the crude ethanolic extract at the concentration of 100, 200, and 300 mg/kg which were dissolved in distilled water by mixing with vortex mixture (MaxiMix^™^ II Vortex Mixer; Thermo Fisher Scientific, Inc., NYSE:TMO).

### 2.9 Induction of diabetes

Forty animals were randomly divided into five groups of eight animals: normal control (NC), diabetic control (DC), *L. macrophylla* 100 mg/kg (LM 100), *L. macrophylla* 200 mg/kg (LM 200), and *L. macrophylla* 300 mg/kg (LM 300). DC and LM animals were supplied 5% fructose solution during the first 2 weeks of the experiment to induce insulin resistance and partial pancreatic β‐cell dysfunction. At the beginning of 3rd week, a low dose of streptozotocin (60 mg/kg BW) dissolved in a citrate buffer (pH 4.4) was injected (i.p.) to DC and LM animals, whereas the NC animals were injected the equal volume of vehicle buffer. One week after the streptozotocin injection, all the animals were measured for nonfasting blood glucose (NFBG) levels by tail‐prick method using a portable glucometer (MicroTech, Zhejiang, China). Animals with an NFBG level ≥16 mmol/L were considered as diabetic. After the diabetes induction, NC and DC animals were supplied normal drinking water, and LM100, LM200, and LM300 animals were, respectively, provided 100 mg/kg BW, 200 mg/kg BW, and 300 kg/kg BW of LM leaf extract during the entire intervention period.

### 2.10 Determination of body weight, Blood glucose, food, and fluid intake

Weekly body weight and blood glucose level and daily food and fluid intake of different animal groups were recorded to assess the effect of intervention.

### 2.11 Oral glucose tolerance test (OGTT)

Oral glucose tolerance test was performed at the 3rd week of the intervention period to measure the glucose tolerating ability of each animal. For OGTT, sugar solution at the dose of 2 g/kg BW was orally administrated to each animal and the blood glucose levels were measured at 0 (just before sugar ingestion), 30, 60, 90, and 120 min after the administration.

### 2.12 Blood and organs collection

After 4 weeks of intervention, animals were sacrificed, collected blood (using heparinated syringe), liver, and pancreas. Collected blood was centrifuged (1100 x g for 15 min at 25–37°C) to separate serum for testing the diabetic parameters (enzymes, insulin, lipid profile, uric acid, creatinine determination, etc.). Livers and pancreas were washed with 0.9% NaCl, dried with tissue paper, and weighed them to keep in a vial containing 10% formalin (formalin must be changed every week till further screening). The liver was used for glycogen measurement and pancreas for histopathological screening.

### 2.13 Analysis of serum and liver glycogen

Serum was analyzed to estimate lipid profile, aspartate transaminase (AST), alanine transaminase (ALT), creatinine kinase (CK‐MB), lactate dehydrogenase (LDH), creatinine, and uric acid levels. Serum insulin was measured by an enzyme‐linked immunosorbent assay (ELISA) method using an ultrasensitive rat insulin ELISA kit (BioVendor, Rat Insulin [TMB] ELISA Kit; Shibayagi Co., Ltd. Gunma, Japan). Liver glycogen concentrations were assessed by a phenol–sulfuric acid method as described by Lo, Russell, and Taylor (1970).

### 2.14 Statistical analysis

Data were presented as a mean ± *SD* of six to eight animals. They were analyzed by statistical software Statistical Package for Social Science (SPSS, version 22.0, IBM Corporation, NY) using one‐way ANOVA followed by Tukey's multiple range post hoc tests. The values were considered significantly different at *p* < .05.

## 3 RESULTS AND DISCUSSION

*Leea macrophylla* is one of the very important herbs in the northern part of Banlgadesh; it is also a known medicinal plant in South Asian territory. This plant has been found to show a number of biological activities which have been expedited for the secondary metabolites present in this plant. This research investigated how the phenolics and flavonoids modulate the pancreatic β‐cell functions controlling other diabetic and diabetic‐related markers in fructose‐fed STZ‐induced type 2 diabetes.

### 3.1 Determination of total phenolic content (TPC)

Total phenolic content of LM ethanol extract was expressed as gallic acid equivalents (GAE) per gram of plant extract. The phenolic content was calculated through the standard curve of gallic acid (y = 23.016x + 0.0282, *R* ^2^ = .9953), and the total phenolic content was found to be 245 ± 5 mg/g dry weight.

### 3.2 Determination of total flavonoid content (TFC)

Total flavonoid content was expressed as quercetin equivalents per gram of the plant extract. TFC was calculated using the standard curve of quercetin (Y = 0.0057x + 0.0524, *R* ^2^ = .9793), and the total flavonoid content was found to be 463.099 ± 5.840 mg/g dry weight.

### 3.3 Food and fluid intake and body weight

**Figure 1:**
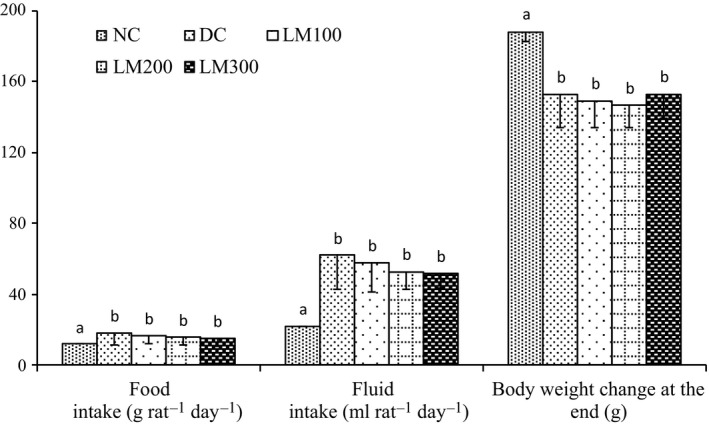
Weekly BW, food, and fluid intake of STZ‐induced rats of each animal group during the experimental period. Data are shown as mean ± *SD* of six to eight animals. ^abc^Values with different superscript letters near the lines for a given week are significantly different from each other group of animals (Tukey's multiple range post hoc test, *p **<** *.05). DC, diabetic control; LM100, *Leea macrophylla* 100 mg/kg BW; LM200; *L. macrophylla* 200 mg/kg BW; LM300 BW, *L. macrophylla* 300 mg/kg BW; NC, normal control

Weekly food and fluid intake and body weight data are presented in Figure 1. The food and fluid intake of the DC group were significantly higher than those of the NC group. Although the food and fluid intake of DC groups were not significantly different from LM groups, it had an average lower fluid intake than DC groups. Fluid intake of NC group was significantly different from that of all other groups. The body weight gain of NC group was significantly higher than all other groups. Usually, STZ‐treated animals show higher consumption of food and water and lower body weight. The lower consumption of food and fluid indicates the improvement of diabetic complications that might be assisted by shorter intestinal transit time and extended gastric emptying time. But *L. macrophylla* treatment groups here had no significant differences in food, fluid, or body weight compared to the DC group.

### 3.4 Determination of weekly blood glucose level

**Figure 2:**
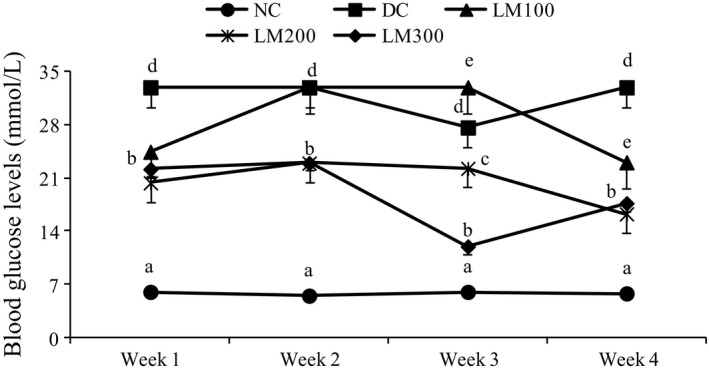
Weekly blood glucose levels of STZ‐induced rats for the whole experimental period. Data are shown as mean ± *SD* of six to eight animals. ^abc^Values with different superscript letters near the lines for a given week are significantly different from each other group of animals (Tukey's multiple range post hoc test, *p **<** *.05). DC, diabetic control; LM100, *Leea macrophylla* 100 mg/kg BW; LM200; *L. macrophylla* 200 mg/kg BW; LM300 BW, *L. macrophylla* 300 mg/kg BW; NC, normal control

Weekly blood glucose change is presented in Figure 2. Nonfasting blood glucose (NFBG) level of both DC and LM groups was very high, but it was started to be reduced gradually after starting treatment with LM. Importantly, LM200 and LM300 reduced the NFBG level very significantly at the 3rd week of intervention, and it was not increased very high until the end of the intervention. But LM100 did not show any significant change in reduction in blood glucose level. However, NFBG levels of NC groups were significantly different from other groups throughout the whole intervention period. Several studies reported that STZ enters insulin‐secreting pancreatic β‐cells through glucose transporter‐2 triggering pancreatic beta cells necrosis and destroys insulin production. This eventually leads to increased blood glucose (Saeed, Deng, & Dai, 2008). This research demonstrates that *L. macrophylla* had a tremendous blood glucose‐lowering effect which required a high dose to observe an effect. This could have been due to the slower absorption of plant extract (Sher, Fakhar‐ul‐Mahmood, Shah, Bukhsh, & Murtaza, 2011). This is consistent with the investigations on the intestinal level by delaying or inhibiting glucose absorption, the peripheral level by facilitating the entry of glucose into cells, and the pancreatic level by stimulating insulin secretion (Hassan, Yam, Ahmad, & Yusof, 2010).

### 3.5 Oral glucose tolerance test (OGTT)

**Figure 3:**
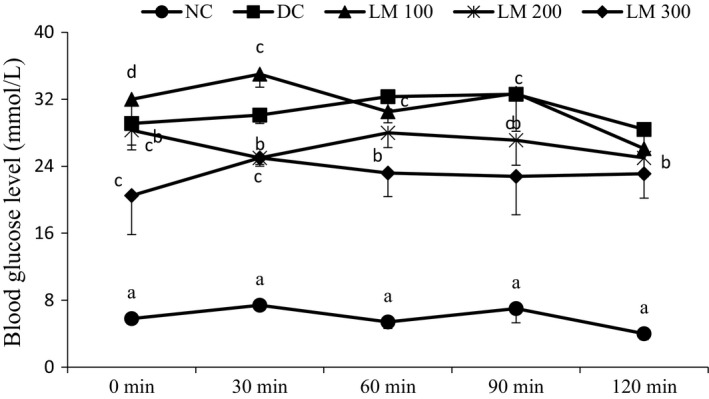
Oral glucose tolerance test (OGTT) of the animal groups at the 3rd week of the experimental period**.** Data are shown as mean ± *SD* of six to eight animals. ^abc^Values with different superscript letters near the lines for a given week are significantly different from each other group of animals (Tukey's multiple range post hoc test, *p **< ***.05). DC, diabetic control; LM100, *Leea macrophylla* 100 mg/kg BW; LM200; *L. macrophylla* 200 mg/kg BW; LM300 BW, *L. macrophylla* 300 mg/kg BW; NC, normal control

Data for OGTT are presented in Figure 3. Oral glucose tolerance showed a drug‐dependent response when performed at the 3rd week of the experiment. Data showed that the glucose tolerance ability of DC group and LM groups was significantly lower than NC group, while a better glucose tolerance ability was observed in LM300 and LM200 groups which showed a significant glucose tolerance at 120 min of OGTT. LM100 hardly defended the glucose load which did not significantly differ from DC group. The oral glucose tolerance test (OGTT) measures the body's ability to use glucose. It is a test of immense significance for using fasting plasma glucose concentration to simplify and facilitate the diagnosis of diabetes. The improved OGTT curve after prolonged treatment with *L. macrophylla* might also be due to the recovery of the pancreas secretion and potentiation of insulin action by LM extracts (Islam et al., 2009). It seems that the lower dose (LM100) is not enough for such insulin secretion and potentiation.

### 3.6 Liver weight, pancreatic weight, relative liver weight, and liver glycogen

**Figure 4:**
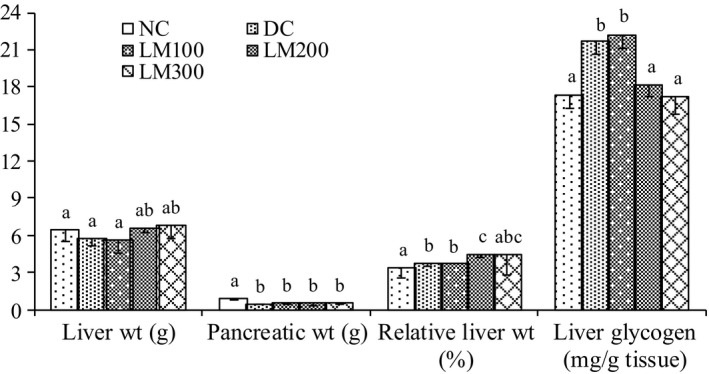
Relative liver weight and liver glycogen for each animal of different groups. Data are shown as mean ± *SD* of six to eight animals. ^abc^Values with different superscript letters near the lines for a given week are significantly different from each other group of animals (Tukey's multiple range post hoc test, *p **< ***.05). DC, diabetic control; LM100, *Leea macrophylla* 100 mg/kg BW; LM200; *L. macrophylla* 200 mg/kg BW; LM300 BW, *L. macrophylla* 300 mg/kg BW; NC, normal control

Results for liver weight, relative liver weight, pancreatic weight, and liver glycogen are shown in Figure 4. No significant difference in the liver weights of NC groups with other groups was observed. Relative liver weights of NC groups were significantly different from DC, LM100, and LM200 groups, but no significant difference was observed between LM300 and NC group animals. Liver glycogen was significantly increased in DC and LM100 groups. Liver glycogen of LM 200 and LM 300 groups was similar to the NC group. Higher pancreatic weight in the treatment groups could be achieved by the regenerative tendency of islets of β‐cells, whereas the degeneration or destruction of insulin‐producing cells may be the leading cause of decreasing the pancreatic weight of DC group (Heidari, Mahmoudzadeh‐Sagheb, & Moudi, 2008; Kim et al., 2006).

### 3.7 Serum enzymes and parameters

Data for ALT, AST, LDH, and CK‐MB are summarized in Table 1. ALT level was significantly increased in DC, LM100, and LM200 groups, but the LM300 level was significantly lower than the other treatment groups while still being elevated as compared to DC. No significant difference of AST level was observed between NC and LM100; however, AST levels of NC group were significantly different from LM200 and LM300 group. LDH levels in DC, LM100, and LM200 groups were significantly different from NC group; however, LDH levels were fully normalized in LM300 group. Similarly, CK‐MB was also found to be normalized in LM300 group. The best dose for serum parameters normalization was found to be LM200. Glycogen serves as a versatile marker to assess insulinomimetic property because it is used as a reservoir for the production of major glycolysis–metabolic fuel, glucose 6‐phosphate, in most of the mammalian cells. And glycogen level severely is decreased in liver due to the insulin resistance in DM (Ramesh & Pugalendi, 2006; Vats, Yadav, & Grover, 2003). LM 200 and LM300 in this study significantly increased the liver glycogen justifying the above phenomena.

**Table 1:** Data for aspartate transaminase (AST), alanine transaminase (ALT), lactate dehydrogenase (LDH), and creatinine kinase (CK‐MB) at the end of the intervention

Group	NC	DC	LM100	LM200	LM300
ALT (U/L)	33.50 ± 4.95^a^	62.00 ± 11.31^b^	142.00 ± 12.73^c^	145.00 ± 22.63^c^	88.00 ± 15.56^d^
AST (U/L)	99.00 ± 8.49a	163.50 ± 0.71^b^	100.00 ± 5.67^a^	129.50 ± 13.54^c^	164.50 ± 3.54^b^
LDH (U/L)	139.00 ± 52.33^a^	276.50 ± 2.12^b^	235.00 ± 21.21^c^	200.00 ± 0.00^d^	109.00 ± 2.80^a^
CK‐MB (U/L)	10.50 ± 6.36^a^	11.00 ± 2.83^a^	23.50 ± 3.54^b^	9.50 ± 6.36^a^	4.00 ± 1.41^ac^

DC, diabetic control; LM, *Leea macrophylla*; NC, normal control. *p* <0.05

Hepatic disorders are evaluated by the level of serum AST and ALT. The increase in ALT and AST in DM usually indicates the hepatotoxic effect of streptozotocin causing liver damage through enzymatic leakage from liver cytosol into bloodstream (Lapshina et al., 2006). Partial restoration of serum ALT levels in our experimental animals explains the contribution of LM extract in lowering the liver markers. However, failure to decrease AST level might be because of inadequate time to control the severe long‐term hyperglycemia resulted from hepatic damage (Lapshina et al., 2006). Apart from these, the active control of *L. macrophylla* in reducing CK‐MB level might be suitable for diabetic cardiopathy resulting an increase in LDH and CK‐MB in diabetic condition (Javad et al., 2012; Jyothirmayi & Kumar, 2011; Zhang et al., 2006).

### 3.8 Serum lipid profile

**Figure 5:**
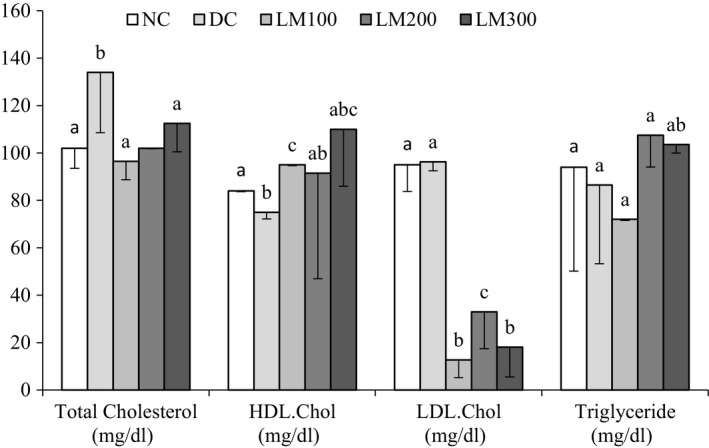
Serum lipid profiles of the animals of different groups. Data are shown as mean ± *SD* of six to eight animals. ^abc^Values with different superscript letters near the lines for a given week are significantly different from each other group of animals (Tukey's multiple range post hoc test, *p **<** *.05). DC, diabetic control; LM100, *Leea macrophylla* 100 mg/kg BW; LM200; *L. macrophylla* 200 mg/kg BW; LM300 BW, *L. macrophylla* 300 mg/kg BW; NC, normal control

Serum lipid status of different animal groups is shown in Figure 5. No significant differences in total cholesterol were observed between NC group and LM groups. DC group has a significantly higher level of total cholesterol which is found to be significantly reduced by LM100. HDL cholesterol was increased, and the highly significant increase was achieved by LM300. LDL cholesterol was extensively reduced in all LM groups, and values were statistically very significant compared to NC and DC group. A significant increase in triglyceride was found between NC and LM200 and LM300 groups. Diabetic hyperlipidemia is one of the major consequences of DM, which causes a lesser transport of glucose in the cells, and therefore, lipids become available as LDL fat deposited to the wall of the artery as fatty plaques transported by HDL to the liver for elimination (Virmani, Burke, & Kolodgie, 2006). Therefore, an increase in HDL and decrease in LDL by LM300 are very expected to be useful for therapeutic application *L. macrophylla*. However, extended research on a high‐fat diet‐induced hyperlipidemia might be effective to find out the inefficiency of *L. macrophylla* to reduce triglyceride and total cholesterol.

### 3.9 Serum insulin, creatinine, and uric acid

**Figure 6:**
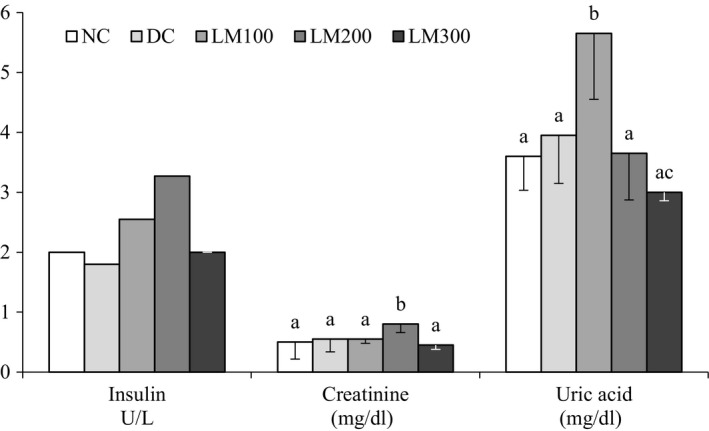
Serum insulin, creatinine, and uric acid profiles of the animals of different groups. Data are shown as mean ± *SD* of six to eight animals. ^abc^Values with different superscript letters near the lines for a given week are significantly different from each other group of animals (Tukey's multiple range post hoc test, *p **<** *.05). DC, diabetic control; LM100, *Leea macrophylla* 100 mg/kg BW; LM200; *L. macrophylla* 200 mg/kg BW; LM300 BW, *L. macrophylla* 300 mg/kg BW; NC, normal control

Data for serum insulin, creatinine, and uric acid are shown in Figure 6. The level of serum insulin concentration of LM100 and LM200 was even higher than NC group. Serum creatinine was lower in the LM300 group compared to all other groups, but the value was statistically significant compared to the NC group. Serum uric acid concentration was significantly higher for LM100 compared to NC. Two diabetic nephropathic markers, serum uric acid and creatinine levels, are induced by diabetic hyperglycemia (Kim et al., 2006). Overproduction of uric acid and creatinine might lead to progressive renal insufficiency, which is also associated with diabetes mellitus, hypertension, hypertriglyceridemia, and obesity (Safi, Mahmood, Khan, & Alhaj, 2004). LM300 treatment group significantly lowers the serum uric acid and creatinine in our experiment suggesting that 300 mg/kg BW of *L. macrophylla* extract have protecting effects of renal impairment.

### 3.10 Pancreatic histopathology

**Figure 7:**
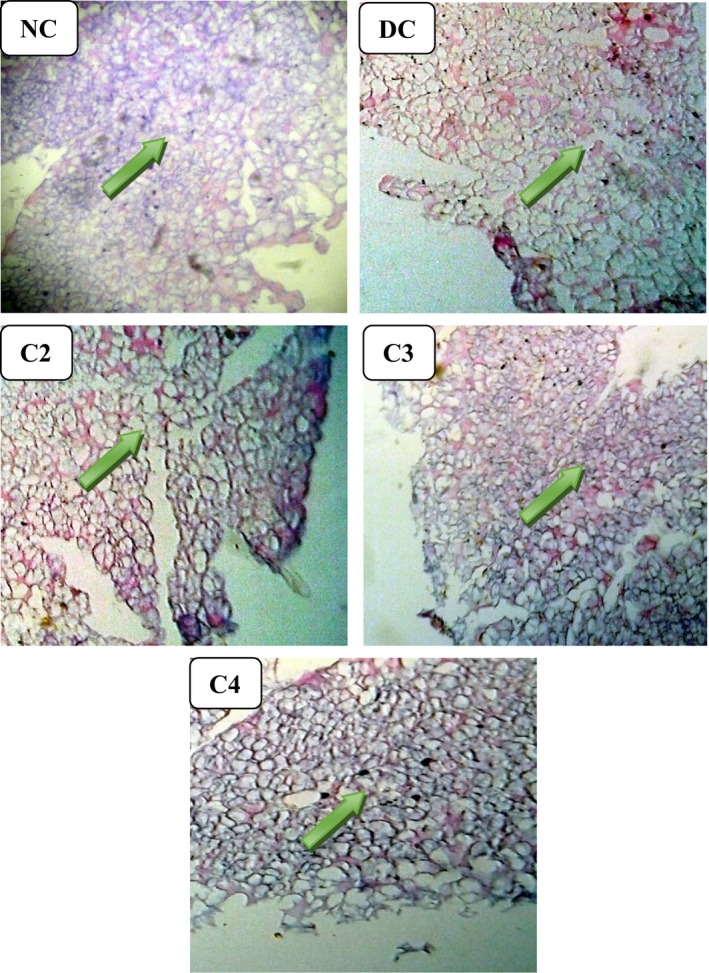
Histopathological examination (magnification 10 × 40) of pancreatic islets at the end of the intervention period. DC, diabetic control; LM100, *Leea macrophylla* 100 mg/kg BW; LM200; *L. macrophylla* 200 mg/kg BW; LM300 BW, *Leea macrophylla* 300 mg/kg BW; NC, normal control. Pancreatic islets in β‐cells are shown by the arrows

Pancreatic tissue sections were used to observe the morphology of pancreases of experimental animals through H&E staining method. Histopathological slides are shown in Figure 7. From the images, it is evident that damaged pancreatic β‐cells were repaired better by LM300 than LM200 and LM100 indicating a consistent effect of LM300 in pancreatic protection to the STZ‐induced type 2 DM.

## 4 CONCLUSION

A plant‐derived drug seems highly attractive for treating diabetes due to the limited efficacy and high risk of adverse effects of synthetic antidiabetic drugs. This research has attempted to establish LM, carrying a marvelous ethnobotanical importance, as a good source of phytomedicinal possibilities to improve the pancreatic health eventually the status of type 2 diabetes. This plant is evident as nontoxic and carries so far very interesting properties in ameliorating the diabetic markers such as insulin and other diabetic‐related markers especially LDL, HDL, LDH, creatinine, uric acid, and CK‐MB, some of which are in animal models. Bioactive compounds isolation and identification other than a primary screening of secondary metabolites are underway to establish a mechanism lying behind the obtained action of *L. macrophylla*.

## CONFLICT OF INTEREST

Authors declare that they do not have any competing interest.
